# Testing inter-observer error under a collaborative research framework for studying lithic shape variability

**DOI:** 10.1007/s12520-022-01676-2

**Published:** 2022-10-01

**Authors:** Lucy Timbrell, Christopher Scott, Behailu Habte, Yosef Tefera, Hélène Monod, Mouna Qazzih, Benjamin Marais, Wendy Black, Christine Maroma, Emmanuel Ndiema, Struan Henderson, Katherine Elmes, Kimberly Plomp, Matt Grove

**Affiliations:** 1 Department of Archaeology, Classics and Egyptology, University of Liverpool, Liverpool, UK; 2 Authority for Research and Conservation of Cultural Heritage, National Museum of Ethiopia, Addis Ababa, Ethiopia; 3 Département Homme Et Environnement, Musée de L’Homme, Paris, France; 4 Institut National Des Sciences de L’Archéologie Et du Patrimoine, Rabat, Morocco; 5 Archaeology Unit, Iziko Museums of South Africa, Cape Town, South Africa; 6 Department of Archaeology, National Museums of Kenya, Nairobi, Kenya; 7 Department of Archaeology, Max Planck Institute for the Science of Human History, Jena, Germany; 8 Mossel Bay Archaeological Project, Western Cape Province, Cape Town, South Africa; 9 Archaeological Studies Program, University of the Philippines, Quezon City, Philippines; 10 Department of Archaeology, Simon Fraser University, Burnaby, British Colombia Canada

**Keywords:** Stone tools, Metric measurements, Geometric morphometrics, 3D printing, Inter-observer reliability

## Abstract

Evaluating error that arises through the aggregation of data recorded by multiple observers is a key consideration in many metric and geometric morphometric analyses of stone tool shape. One of the most common approaches involves the convergence of observers for repeat trails on the same set of artefacts: however, this is logistically and financially challenging when collaborating internationally and/or at a large scale. We present and evaluate a unique alternative for testing inter-observer error, involving the development of 3D printed copies of a lithic reference collection for distribution among observers. With the aim of reducing error, clear protocols were developed for photographing and measuring the replicas, and inter-observer variability was assessed on the replicas in comparison with a corresponding data set recorded by a single observer. Our results demonstrate that, when the photography procedure is standardized and dimensions are clearly defined, the resulting metric and geometric morphometric data are minimally affected by inter-observer error, supporting this method as an effective solution for assessing error under collaborative research frameworks. Collaboration is becoming increasingly important within archaeological and anthropological sciences in order to increase the accessibility of samples, encourage dual-project development between foreign and local researchers and reduce the carbon footprint of collection-based research. This study offers a promising validation of a collaborative research design whereby researchers remotely work together to produce comparable data capturing lithic shape variability.

**Supplementary Information:**

The online version contains supplementary material available at 10.1007/s12520-022-01676-2.

## Introduction

Shape analyses are becoming an increasingly popular methodology for examining lithic variability in the archaeological record. As such, traditional linear metrics and geometric morphometrics (GMM) are often employed to capture morphological information on stone tools (Cardillo 2010; Lycett and von Cramon-Taubadel 2015; Matzig et al. 2021). Combining morphological data from multiple observers is frequently necessary in studies of lithic assemblages, to increase sample size and/or to perform inter-site/inter-assemblage analyses, yet this can be problematic due to the possibility of introducing inter-observer error into the data (Lyman and VanPool 2009). Such error has multiple potential sources, can be introduced at various stages in the workflow, and can skew results by obscuring any “real” signals in the data (Fruciano 2016); examining the magnitude of inter-observer error is therefore imperative to validate whether meta-analyses are robust. International researchers are increasingly being encouraged to work collaboratively in order to remotely produce archaeological and anthropological datasets (Chang and Alfaro 2015; O’Leary and Kaufman 2011; Scerri et al. 2020; Timbrell 2020, 2022)—in some case even crowdsourcing morphometric data (Chang and Alfaro 2015). However, it is frequently impossible for observers to converge on the same material to record repeat trials for an inter-observer repeatability assessment. Such control tests therefore need to be appropriate for the specific research design, and customized solutions for evaluating error under collaborative research frameworks should be developed (Fruciano 2016). Here, we present an innovative analysis of inter-observer error involving the compilation of standardized photographs and measurements of lithics from multiple observers for metric and GMM analysis (Timbrell 2022).

Traditionally, lithic shape variation has been examined through qualitative descriptions (Inizan et al. 1999), typological classification (Bordes 1961) and/or linear measurements (Roe 1964; McNabb 2017). Advancements in biological morphometrics and computing have meant that geometric morphometrics are now also routinely applied in the analysis of lithic morphologies (Bookstein 1991; Buchanan et al. 2018; Cardillo 2010; Lycett 2009; Serwatka and Riede 2016). GMM approaches are split into methods that use landmarks and outlines, the former representing shape through homologous points (landmarks) superimposed on a two-dimensional (2D) or three-dimensional (3D) object and the latter applying geometric descriptions of homologous outlines or surfaces (Mitteroecker 2021). Landmark-based methods allow for specific aspects of morphology to be captured without the inclusion of random noise (i.e. shape dimensions that are not pertinent to the research question); however, their application to certain non-biological structures, such as lithics and other archaeological artefacts, is often more difficult as the identification of homologous landmarks can be subjective (Okumura and Araujo 2018). Outline-based GMM, on the other hand, avoids certain issues of homology through quantifying the gross shape of each specimen (Klingenberg 2008), making them ideal for describing shape variation of lithics in archaeological studies (e.g. Iovita 2009, 2011; Ivaonovaité et al. 2020; Matzig et al. 2021; Mesfin et al. 2020; Wang and Marwick 2020).

Assessment of the levels of inter- and intra-observer error under different methodological approaches to studying lithic shape is vital, and several studies have examined error in metric and GMM analyses at different phases of the workflow (Evin et al. 2020; Fagerton et al. 2014; Lyman and VanPool 2009; Macdonald et al. 2020; Menedez 2017; Osis et al. 2015; Perini et al. 2005; Robinson and Terhune 2017; von Cramon-Taubadel et al. 2007; Yezerinac et al. 1992). Problematic landmarks, i.e. those that are difficult to consistently locate, can be a source of error in landmark-based GMM analysis (Fagerton et al. 2014; Menedez 2017; Robinson and Terhune 2017; von Cramon-Taubadel et al. 2007), even for experienced observers (Chang and Alfaro et al. 2015). von Cramon-Taubadel et al. (2007) found that repeating the digitization procedure was the most suitable method for assessing the precision of landmarks, with adequate landmark definitions imperative for reducing error. Yezerinac et al. (1992) also found that ill-defined measurements were a factor increasing error in metric data; in addition to operator experience, the precision of the measuring device and the conditions under which the measurements are made, such as lighting. Combining metric measurements from more than one observer, therefore, is likely to be suitable only when the dimensions are standardized and easily measured, and the conditions, the precision and quality of the equipment and the technique of recording the data are comparable (Lyman and VanPool 2009).

Comparatively, fewer studies have examined the levels of inter-observer error in outline-based GMM methods. Evin et al. (2020), in an investigation of error between morphometric approaches, found that although methods that employ landmarks were the most sensitive to error, outline data saw relatively lower levels of intra-observer error compared to inter-observer error, with photography being an influential source of variance between observers. Digital photography is widely used in 2D GMM as it is inexpensive, easy to perform and does not require extremely specialist knowledge or equipment, with the digitization of landmarks and/or outlines on the resulting images providing a 2D representation of the 3D object. The focal length and specifications of the lens used can, however, cause parallax error; the optical distortion that occurs when the specimen is too close or not directly centered beneath the lens (fisheye). Nonetheless, several studies employing both landmark and outline methods suggest that 2D GMM data are minimally affected by parallax error, especially when the camera set-up is standardized and calibrated, with deviations small and constant enough for accurate analyses (Caple et al. 2018; MacDonald et al. 2020; Mullin and Taylor 2002; Riano et al. 2009). Overall, outline-based methods are likely more suitable for collaborative research designs in studies of lithic shape; due to the objectivity of data capture, the fact that landmark methods have high rates of inter-observer error, though this is more pertinent during landmark digitization than object photography (Evin et al., 2020), and the potential to reduce inter-observer error through the standardization of the photography procedure.

Although the inter-observer error is a concern in any collaborative research design, collating data from multiple observers is often necessary in archaeological research, be it to increase sample sizes, facilitate interdisciplinary research and/or enable access to disparate data (Timbrell 2020). The latter is especially important when considering issues of income-disparity, childcare and disability that can disproportionately disadvantage researchers who are unable to travel extensively to collect data. Global catastrophes, such as pandemics, climate change and conflict, can also temporarily delay international research through the constraints imposed on travel and safety, requiring researchers to develop scientifically sound remote models of data generation (Scerri et al. 2020). Timbrell (2022) presents such a framework, which involves the documentation of lithic shape by multiple collaborators. These types of approaches have additional benefits for decreasing the carbon footprint associated with accessing multiple international samples and fostering knowledge-sharing through dual project development and the division of responsibilities so that both foreign and local researchers take on principal roles within a given project, which is particularly crucial across the Global North–South divide (Chirikure 2015; Douglass et al. 2020; Else, 2022). Indeed, collaborative approaches accord with the open science initiative in archaeology, which advocates that data stewardship should be centered around researchers collecting and sharing data on behalf of the scientific community, as opposed for the betterment of a single individual’s career (Marwick et al. 2017).

While collaborative data collection offers a promising new framework for generating and sharing data internationally, the analysis of inter-observer error is imperative to validate such an approach. Here, we present a unique control test that involves the production of 3D printed replicas of a lithic reference collection, which can be distributed among observers and measured following the same protocols used to collect the actual data. We then examine the differences between the datasets, knowing that each collaborator has recorded the same data from identical copies of the artefacts. Using this approach, we evaluate whether the compilation of data from multiple observers is conducive to error, and thus could negatively bias the results of a collaborative study.

## Materials

**Fig. 1:**
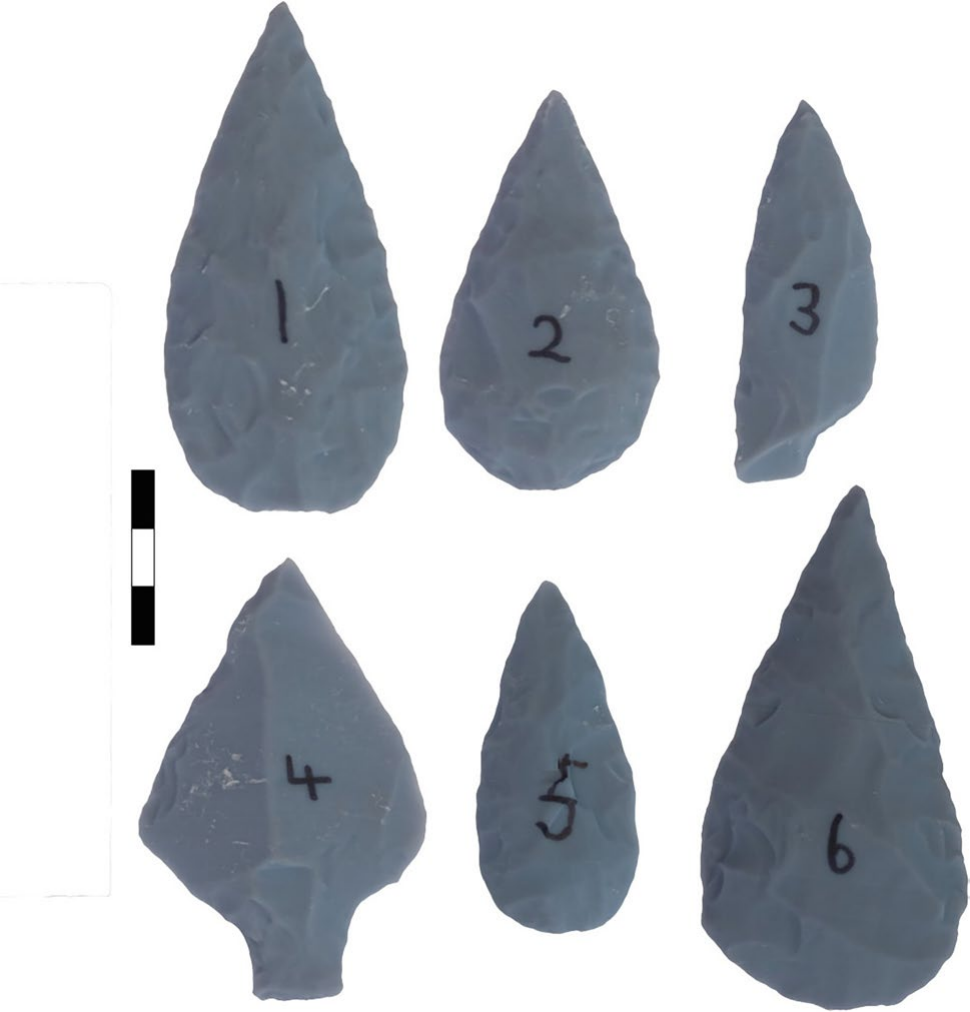
The six 3D printed replica tools. Original lithics were knapped and scanned by CS in preparation for 3D printing. Example photos were taken by SH. *Scale* = 3 cm

Six lithic points were knapped using fine-grained flint from Caistor Quarry, Caister St Edumunds, UK and scanned for 3D printing at the University of Liverpool (Fig. 1). The reference tools varied in both size and shape, encapsulating a range of morphologies characteristic of the empirical sample to be studied in the main project (African Middle Stone Age assemblages). While flint is not a feature of African lithic assemblages, it could be considered representative of the finer-grained materials, such as obsidian, chert and heat-treated silcrete, exploited during the Middle Stone Age (Key et al. 2021; Sahle et al. 2013). The tools were produced on flakes and retouched using: (1) direct freehand hard hammer percussion (quartzite hammerstones), (2) direct soft free-hand hammer percussion using an antler hammer and (3) handheld pressure flaking using an antler tine supported in a tanned leather pad. Each tool was colored blue using craft enamel spray paint to aid scanning.

Next, each lithic was scanned with a freshly calibrated Einscan Pro 2X structured light scanner with a colour camera, using the combined feature and texture mapping in the high-resolution setting. Initial scans were performed with the lithics placed vertically in a foam holder using fixed scan mode aligned with an automated turntable and coded targets (scans taken every 11.25 degrees, i.e. 32 scans). The models were then completed by switching to the “align by” feature using the turntable (32 scans), and the lithic was rescanned (2–3 times) until a complete model was achieved. All alignment was automatic to produce a watertight mesh; no holes were filled. Each model was sharpened using the Einscan high-setting and saved as.obj files without decimation (see Supplementary Online Table 1 for further data on each model).

**Fig. 2:**
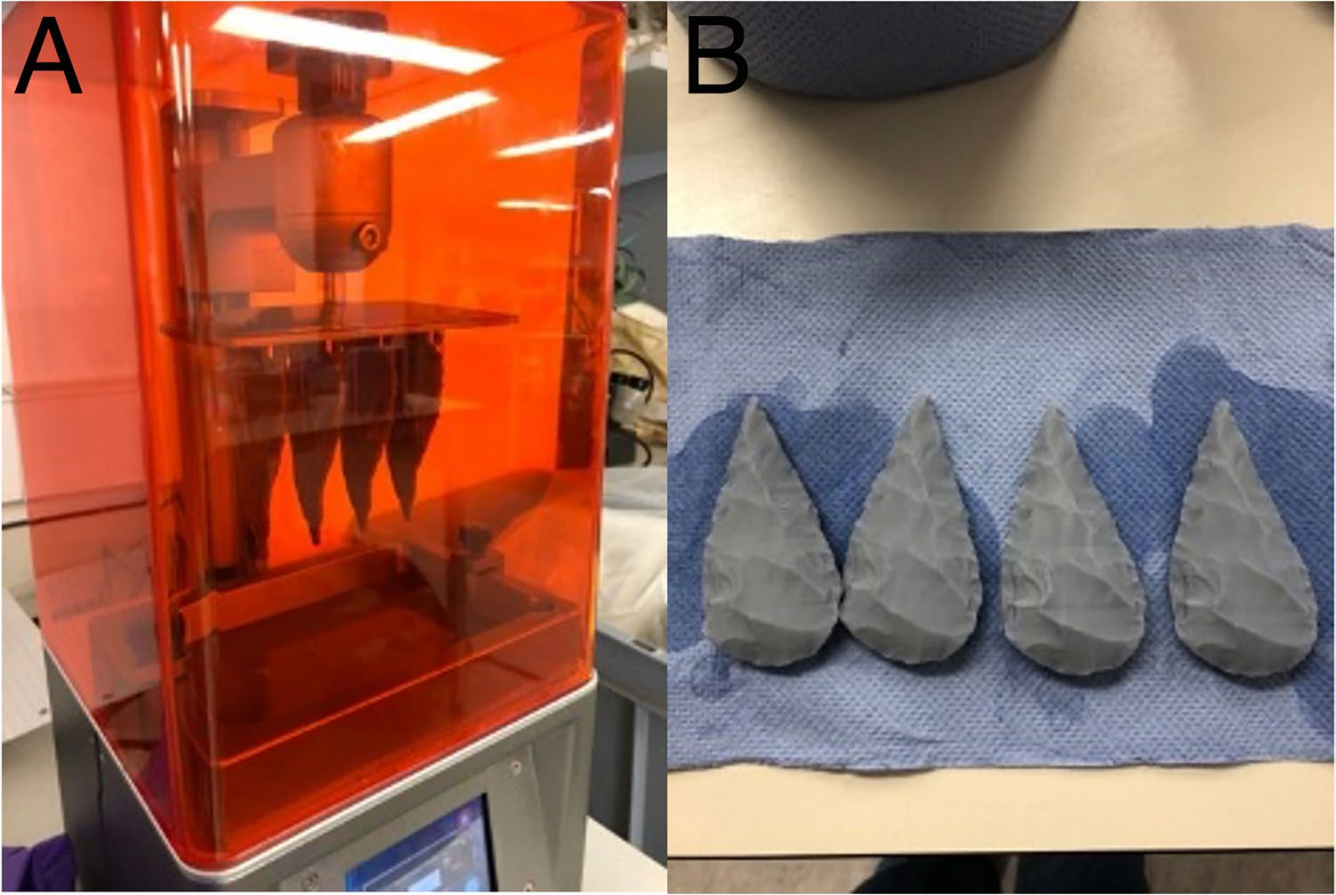
Photographs from the 3D printing process. **A** The 3D model of the tool is sent to the machine for printing. **B** The resulting 3D prints once removed from the supports are cleaned using ethanol. 3D printing was carried out by LT and CS

The 3D models were processed for printing using Chitubox v1.8.1. Medium-sized automated supports were applied using this software at 90% total coverage to provide a strong foundation for the 3D prints. We used an Elegoo Mars 2 Resin printer, with a new printer film, using standard grey Elegoo LCD UV curing 405 Nm photopolymer resin with recommended Elegoo settings (Fig. 2). The prints were extracted from the print bed, and the supports were removed by hand prior to being rinsed in ethanol and cured in direct sunlight. Each tool was printed six times to create six copies of the assemblage, resulting in 36 prints in total.

## Methods

Each tool was assigned a number (Tool 1–6; Fig. 1), and a replica copy of the assemblage was sent to researchers at six independent institutions (Table 1). Data collection protocols, outlined in detail by Timbrell (2022) and described in Supplementary Online Resource S1, were developed to standardize the documentation of lithic shapes through photography and measurements. These procedures were followed by all observers across the study to produce equivalent data. Instructions for object position, camera position and settings and lighting were specified and tightly controlled (Supplementary Online Resource S1). In addition, a scale (sourced in situ by the observers) was placed in each photograph to ensure a measure of size was recorded. Table 1 reports the camera and lenses used to capture each replica assemblage in the study; high-quality equipment was accessed by all observers either through their institution directly or through funding provided by the project. Three basic measurements on each tool were also taken to record morphological length, width and thickness (see Supplementary Online Fig. 1 for a schematic) at a resolution of 0.1 mm. We defined length as the maximum dimension of the point, width as the maximum measurement in the perpendicular dimension to length and thickness as the maximum measurement in the third dimension, following Shea (2020).

Prior to distribution among institutions, all 36 replicas were also recorded by a single observer (LT) to produce a comparative dataset. Photography was performed using a Canon M50 camera with an EF-S 60 mm f/2.8 Macro USM lens and the three measurements were taken using digital calipers. This enabled us to determine the magnitude of intra-observer measurement error, for comparison with the magnitude of inter-observer error, had the project been carried out by a single individual under a traditional research framework.

Data were uploaded onto a communal data sharing platform (Google Drive) by each observer for processing and analysis by a single observer (LT). Analyses were performed in the R software environment (R Core Team 2020). Data and code can be found on the GitHub repository for the project: https://github.com/lucytimbrell/error_analysis_lithics/.

**Table 1:** Summary of the observers and the photography equipment used. This equipment was sourced locally; in most cases, the institutions already had access to the necessary apparatus; however, in some cases, it was rented and/or purchased and donated to the institution after the project, following guidelines provided by The Wenner Gren Foundation

Assemblage number	Institution	Abbreviation	Country	Camera body	Camera lens
1	Institut National des Sciences de l’Archéologie et du Patrimoine	INSAP	Morocco	Nikon D7100	Nikon AF-S Micro Nikkor 105 mm
2	Iziko Museums of South Africa	IM	South Africa	Canon 6D II	Canon 100 mm 2.8 Macro
3	Mossel Bay Archaeological Project	MBAP	South Africa	Nikon D300s	Nikon AF Micro Nikkor 60 mm 1:2.8D
4	National Museum of Ethiopia	NME	Ethiopia	Canon EOS DSLR 200D	Canon Tamron 60 mm Macro Di II
5	National Museums of Kenya	NMK	Kenya	Nikon D5300	Nikon AF-S Micro Nikkor 40 mm
6	Musée de l’Homme	MH	France	Nikon D5200	Nikon AF-S Nikkor 24–70 mm

### Metric analyses

We first computed the intra-class correlation coefficient ($ICC$) using the “psych” R package (Revelle, 2022) to assess the agreement between the six observers in measuring the six tools for length, width and thickness. The $ICC$ compares the variability within repeat measurements whilst contrasting variability between groups of measurements (Barlett and Frost 2008; Fruciano 2016; Koo and Li 2016; Shrout and Fleiss 1979). Specifically, we used a two-way mixed effects model to compute the $ICC$, with the set of observers considered a fixed effect. To assess the reliability of data collection, we next calculated and compared the mean, variance, technical error of measurement ($TEM$) and percentage technical error of measurement ($\% TEM$). The mean and variance (expressed as the standard deviation) were calculated for each measurement on each tool, with the $TEM$ and $\% TEM$ calculated to compare pairs of observers across all measurements on all tools. The $TEM$ reflects measurement precision between observers, and is calculated as:

$$TEM = \sqrt{\frac{\left({\sum }_{1}^{N}\left({\sum }_{1}^{K}{M}^{2}\right)\right) - \left({\left({\sum }_{1}^{\mathrm{K}}M\right)}^{2}/K\right)}{N\left(K-1\right)}}$$

where $\mathrm{N}$ is the number of subjects, $\mathrm{K}$ is the number of observers, and $\mathrm{M}$ is the measurement (modified from Ulijaszek and Kerr [1999]). The *%TEM* represents the magnitude of the error as a percentage of the mean of the measurement/variable studied. It is calculated as:

$$\%TEM = 100 (\frac{TEM}{\overline{v} })$$

where $\overline{v }$ is the average value of the raw measurements, taken across all measurements on all tools by multiple observers. The values obtained for these metrics must be subjectively assessed according to the research question, as there is no standard applied threshold of error deemed to be “acceptable”. Following Lyman and VanPool (2009)’s analyses of projectile points, we propose that a $\%TEM$ of < 4 could be an acceptable level of error without negative consequences on the results. Lastly, we calculated the coefficient of reliability $(R)$, which ranges from 0 to 1, with 1 indicating very high congruence between measures. We used the following formula outlined in Lyman and VanPool (2009):

$$R = {\sigma }_{v}^{2}/\left({\sigma }_{\mathrm{v}}^{2} + {\sigma }_{\mathrm{d}}^{2}\right)$$

where ${\sigma }_{\mathrm{v}}^{2}$ is the variance of all raw measurements on all tools taken by two observers and ${\sigma }_{\mathrm{d}}^{2}$ is the variance of the difference between those two sets of measurements. Similarly to the $ICC$, the coefficient of reliability distinguishes between the variability between the specimens and that which results from random measurement error. However, whilst $R$ can only be calculated between pairs of observers, the $ICC$ represents an overall metric for measurement error across all observers.

Random error can inflate the amount of variance within a sample, resulting in a loss of statistical power as noise obscures true differences in means (Fruciano 2006; Yezerinac et al. 1992). To evaluate the levels of error in the multiple observer data in relation to the single observer data, we used two-sample t-tests to compare differences in mean and F-tests to compare differences in variance. If there is high inter- and/or intra-observer error, variation within replicas of the same tool will be increased and differences in the mean values for each tool will be significantly different.

### Two-dimensional geometric morphometric analysis

In preparation for GMM analysis, each image was processed using the “object select’ tool in Adobe Photoshop, which automatically determines the contour of the object. Once the contour was highlighted, the object was filled with solid black to help facilitate the extraction of outline data. All processed images were then synthesized into a single thin-plate spline (.tps) file using tpsUtil, and the outline data were extracted using tpsDig2. The outline of each artefact was represented by an average of 2856 equidistant points, which were scaled through the specification of the pixel-to-centimeter ratio for each image (see Supplementary Online Fig. 2 for a visualization of the data). The outline data were saved as (x, y) coordinates within the.tps file and imported into R.

**Fig. 3:**
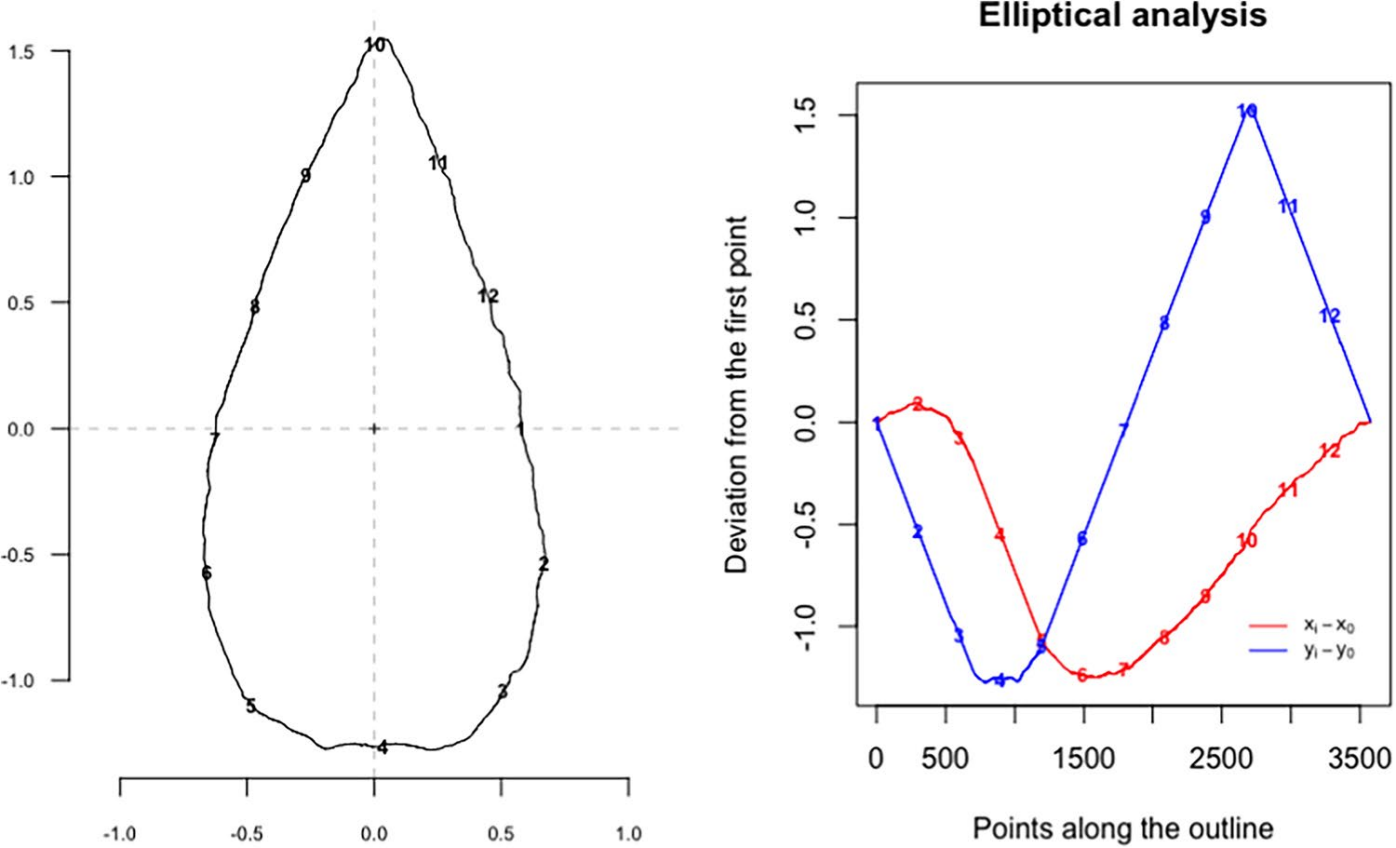
A schematic of the Elliptic Fourier fitting process that generates the raw shape data for geometric morphometrics. Coefficients of sine and cosine terms (harmonics) are computed to reconstruct the x (blue) and y (red) coordinates from an arbitrary starting point moving along the outline

Using the “Momocs” R package (Bonhomme et al. 2014), the outlines were standardized following Bonhomme et al. (2017) by normalizing to a common centroid, scaling to centroid size and aligning along the long axis of the object. We then performed elliptic Fourier analysis (EFA) to convert the geometric data to frequency data, with the outline decomposed into a series of repeating trigonometric functions, referred to as harmonics (Caple et al. 2017; Fig. 3). The appropriate number of harmonics were identified to capture sufficient information on shape; this was deemed to be 8 harmonics, achieving 99% harmonic power (Caple et al. 2017).

Next, we performed a principal components analysis (PCA) on the elliptic Fourier coefficients to reduce the dimensionality of the data. Principal components (PCs) are constructed to highlight the main axes of morphological variance (Zelditch et al. 2004). Like with the metric data, we calculated the $ICC$ and $R$ values to partition the variance from the inter-observer error for the PC scores of repeat captures (Daboul et al. 2018; Fruciano 2006). Due to the nature of PC scores, we were unable to obtain an informative relative measure of dispersion ($\%TEM)$ and instead refer to the standard deviation (calculated as the square root of the variance) as absolute measures of dispersion. This is because, when the mean of a set of repeat captures falls close to the mean of a PC (~ 0) and has a low standard deviation (~ 0), the %TEM would be very high despite the tight clustering of the repeated measures along that PC. In addition, we applied linear discriminant analysis (LDA) to the PC scores, with the equal sample sizes used as the prior group probabilities (1/6) of a repeat belonging to a certain group based on their outline shape alone (Mitteroecker and Bookstein 2011). In this analysis, we tested firstly whether the tools could be distinguished based on their shape alone, and then whether the observers could be identified. One would expect high classification results when discriminating between tools and low classification results when discriminating between observers if inter-observer error is low.

## Results

### Linear metric analysis

**Fig. 4:**
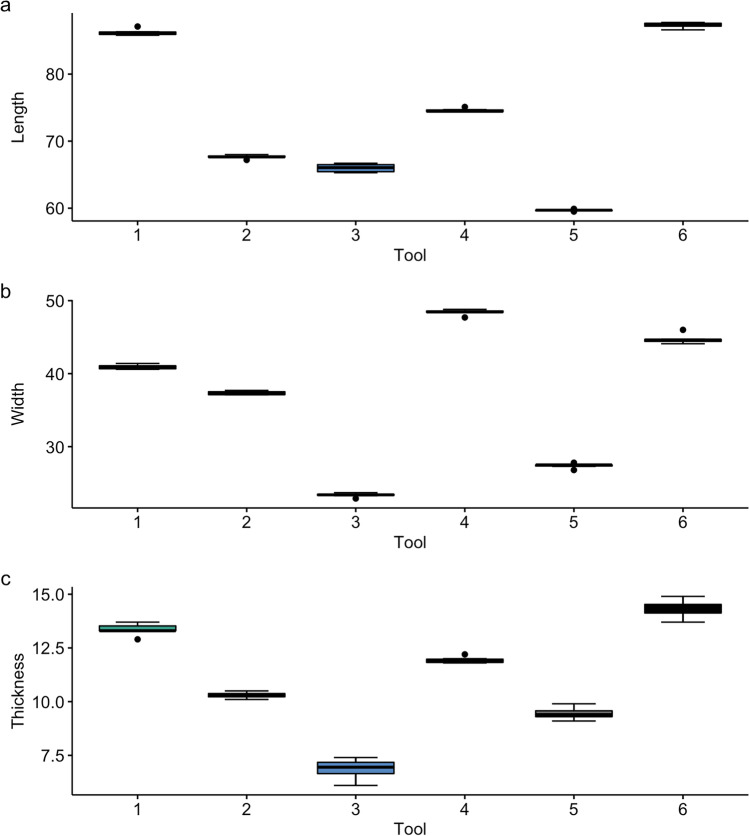
Boxplots demonstrating the distribution of length, width and thickness (mm) collected by multiple observers for each tool (1–6)

We first explored whether the measurements were recorded consistently on the replicas between observers. Figure 4 shows the distribution of the multiple observer data through boxplots; most of the measurements have very limited variance around the mean, and all tools were significantly different to each other across all measurements when tested using Tukey’s honestly significant difference (HSD; *p* < 0.001). Thickness is the most variable dimension recorded, probably because it is more difficult to orient the tool for this measurement than it is for length or width. Calculation of the coefficient of reliability between each pair of observers found that all values of $R$ were > 0.999, suggesting that over 99% of the variance in each measurement is due to variability between the specimens as opposed to error. We calculated the $TEM$ as 0.368 and the $\%TEM$ as 0.908, supporting that less than 1% of the variance in the dataset is related to measurement error. Finally, the $ICC$ score confirmed that there is a very high absolute agreement between the observers ($ICC$ = 1, *p* < 0.001).

We then compared the measurements taken by multiple observers with those taken by a single observer as a means of comparing intra- and inter-observer errors. We first calculated the coefficient of reliability for the single observer for each pair of replica assemblages—we found that all values were > 0.999, indicating very high congruence between repeat captures by the single observer. Table 2 reports the mean and standard deviation of length, width and thickness for the single observer compared to multiple observers; two-sample t-tests found that there were almost no statistically significant differences in means between the data sets (1/36 = *p* < 0.05; Table 3). However, F-tests found that half of the measurements show statistically significance differences in variance, particularly along length and width (Table 3). This demonstrates that the single observer is generally less prone to error, which is likely due to a combination of the familiarity of this observer to both the metric definitions and the assemblage and the fact that the same equipment was used to measure all of the replicas. Nonetheless, the fact that these differences in variance only resulted in a single instance of significant difference in mean, plus the standard deviation does not exceeds 0.7 mm, suggests that the effects of inter-observer error are minimal on the results.

**Table 2:** Summary statistics reporting the mean (m) and standard deviation (sd) obtained for length, width and thickness, recorded by multiple observers versus a single observer for each tool (1–6). Standard deviation values have been rounded to 3 decimal places

Tool	Length (mm)				Width (mm)				Thickness (mm)			
	Multiple		Single		Multiple		Single		Multiple		Single	
	m	sd	m	sd	m	sd	m	sd	m	sd	m	sd
1	86.2	0.471	86.3	0.175	40.9	0.308	40.9	0.103	13.4	0.281	13.3	0.248
2	67.6	0.266	67.6	0.089	37.3	0.258	37.5	0.228	10.3	0.141	10.4	0.075
3	66.0	0.613	66.3	0.137	23.4	0.266	23.3	0.225	6.87	0.472	6.72	0.075
4	74.6	0.279	74.4	0.299	48.4	0.374	48.5	0.103	11.9	0.151	11.8	0.105
5	59.7	0.133	59.7	0.075	27.4	0.335	27.6	0.082	9.45	0.281	9.48	0.147
6	87.3	0.405	87.4	0.063	44.7	0.659	44.6	0.126	14.3	0.415	14.2	0.117

**Table 3:** *P*-values from t-tests (difference in mean) and F-tests (difference in variance) comparing the metrics (length, width and thickness) for each tool (1–6) measured by multiple observers versus a single observer. Statistical significance (*p* < 0.05) is marked by an asterisk (*). All values have been rounded to 3 decimal places

Tool	Length (mm)		Width (mm)		Thickness (mm)	
	T	F	T	F	T	F
1	0.815	0.049*	0.632	0.032*	0.673	0.792
2	0.678	0.032*	0.264	0.792	0.240	0.193
3	0.342	0.005*	0.498	0.724	0.475	0.001*
4	0.342	0.879	0.689	0.013*	0.037*	0.446
5	0.608	0.238	0.152	0.008*	0.804	0.182
6	0.575	0.001*	0.646	0.002*	0.653	0.015*

### Geometric morphometric analysis

**Fig. 5:**
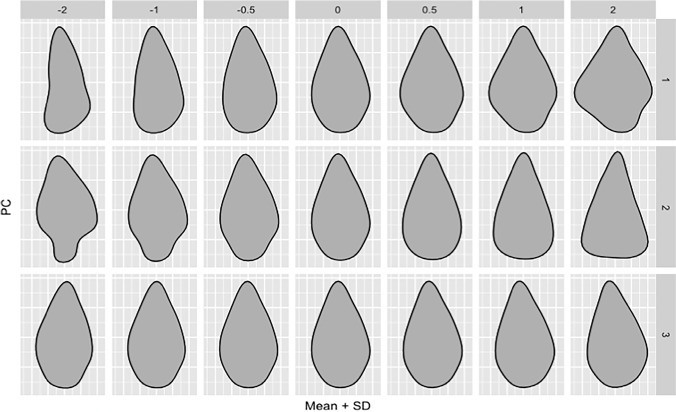
Principal component (PC) contributions along the first 3 axes of variance within the multiple observer outline data

PCA was used to highlight variance in the multiple observer data. The first 3 PCs represented > 90% of the variation between the replicas, and thus were explored in this study. Figure 5 demonstrates the shape differences highlighted by PC1-3. PC1 represents 59.7% of the total variance, whilst PC2 and PC3 account for 33.4% and 3%, respectively (see Supplementary Online Fig. 3 for scree plot of PC loadings and cumulative variance).

**Fig. 6:**
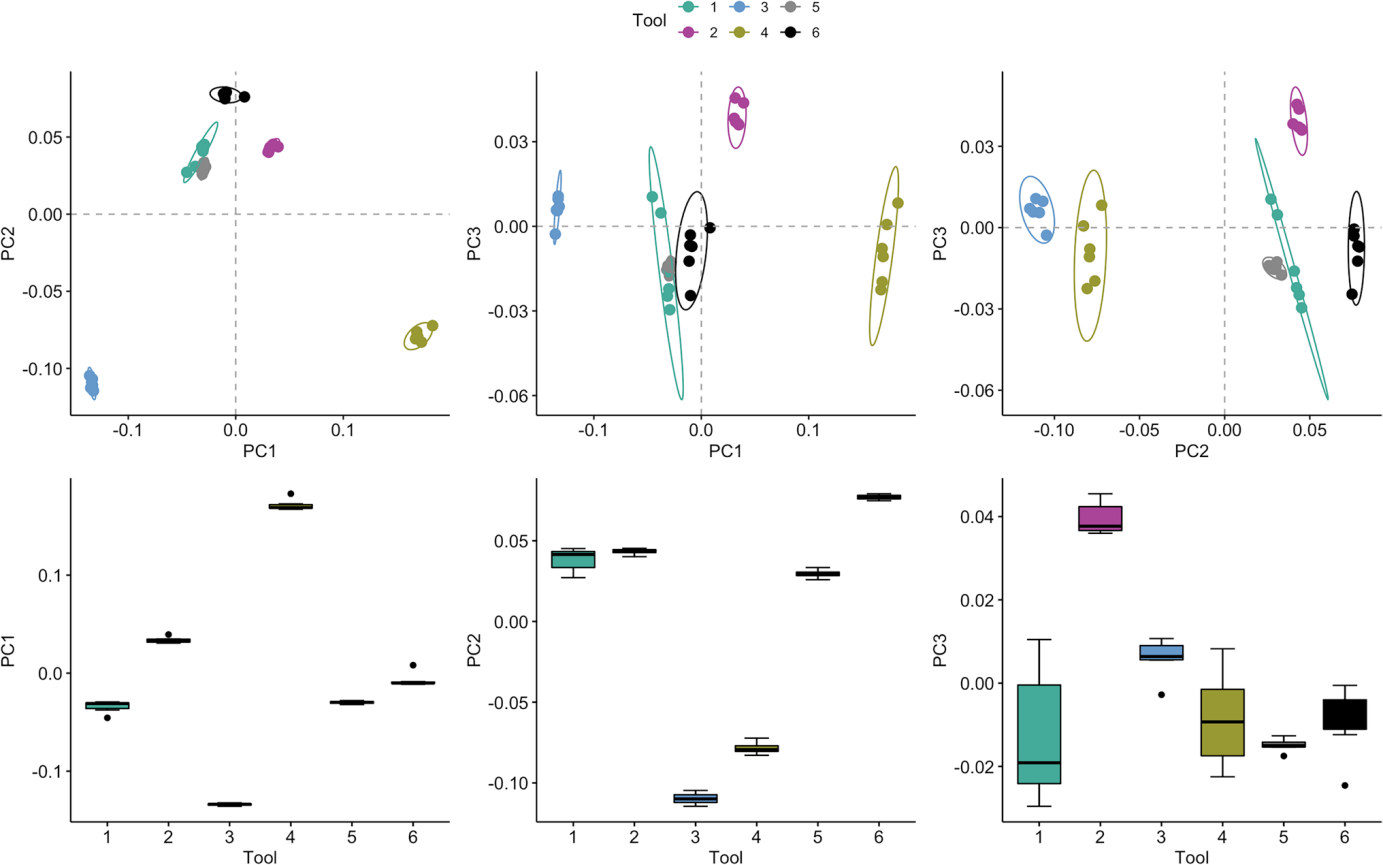
Scatterplots (top row) and boxplots (bottom row) of repeat capture scores along principal components (PC) 1–3, demonstrating the clustering within tools (1–6). PC1 represents 59.7% of the total variance, whilst PC2 and PC3 account for 33.4% and 3%, respectively

When the first 3 PCs are plotted against each other, clear clustering occurs, demonstrating that replicas of the same tool tend to share more similarities than that of different tools (Fig. 6). However, there is notable variation within tools along PC3, suggesting that inter-observer error deriving from photography equipment and set-up is prevalent in this dimension. PC3 is an axis of variation represented by slight asymmetries in convexity at the proximal end (Fig. 5), thus likely reflecting parallax error between observers. Additionally, we note some overlap between certain tool groups, although this is primarily because these tools share similar shapes once size is removed (Supplementary Online Fig. 2). For example, Tool 5 sometimes plots within the range of variation for Tool 1 and only shows statistically significant differences in mean from this tool along PC2 (*p* < 0.008; see Supplementary Online Table 2 for Tukey’s HSD results comparing differences in mean between tools). To tease apart the variation between the tools and that associated with the error, we calculated the coefficient of reliability between each pair of observers, which ranged between 0.960 and 0.999 (Table 4), suggesting that < 4% of the variance is due to inter-observer error, which lies within our acceptable threshold. The $ICC$ was computed using the first 3 PC scores to determine levels of similarity between the six observers, whilst taking into account the variability between the tools, and found an almost perfect agreement ($ICC$ = 0.99, *p* < 0.001). Finally, we found that an LDA could discriminate accurately between the replica groups (94% classification accuracy) and could not differentiate between observers (0% classification accuracy).

**Table 4:** Coefficient of reliability ($R$) values for pair-wise combinations of observers using the first 3 PC scores. For observer abbreviations and associated assemblage numbers, see Table 1. All values have been rounded to 3 decimal places

	INSAP	IM	MBAP	NME	NMK
IM	0.988				
MBAP	0.978	0.960			
NME	0.984	0.975	0.995		
NMK	0.969	0.969	0.985	0.992	
MH	0.989	0.978	0.993	0.999	0.990

**Fig. 7:**
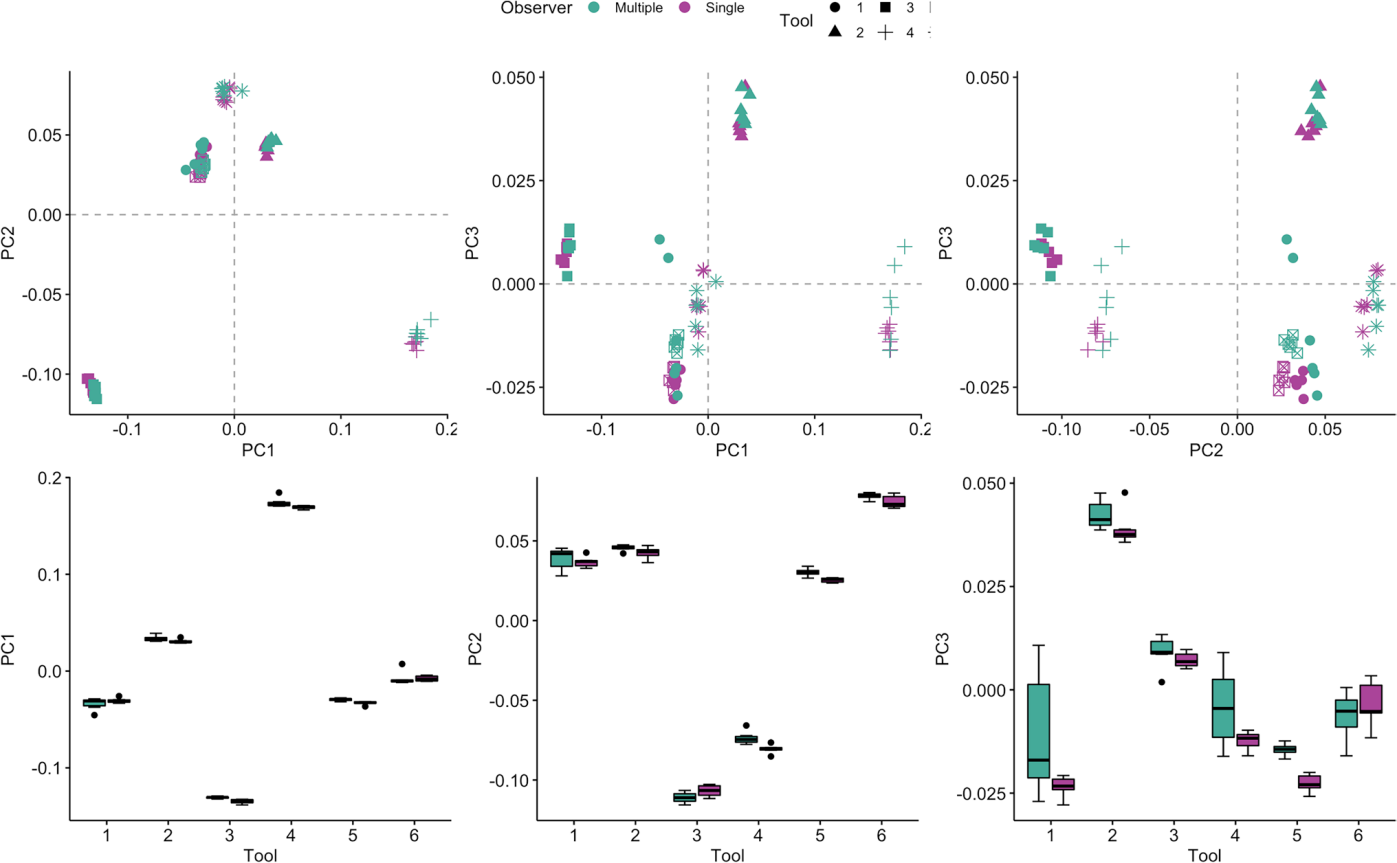
Scatterplots (top row) and boxplots (bottom row) of repeat capture scores along principal components (PC) 1–3, demonstrating the clustering within tools (symbols) and between data sets (colors). PC1 represents 60.4% of the total variance, whilst PC2 and PC3 account for 33.5% and 3.3%, respectively

Next, we compared the levels of error obtained when collating photographs from multiple observers and that which arises when all replicas are photographed by the same observer. We performed another PCA with data acquired from both sets of images (see Supplementary Online Figs. 4-5 for PC contributions and loadings) and produced scatterplots of PC1–3. Figure 7 demonstrates clear clustering between tools recorded in both sets of data along PC1 and PC2. However, along PC3 there is clear variability within repeats when grouped by the observer (multiple vs single). F-tests found that the variance among certain tools was only significantly higher for the multiple observers in three cases, i.e. tool 4 and 1 along PC3 and tool 4 along PC1 (Table 5). Two-sample t-tests found statistically significant differences in means between the data sets, but these are limited (5/36 = *p* < 0.05; Table 5). Table 6 and Fig. 7 demonstrate that the data collected by a single observer returns lower variance, though this pattern is not strong, and, in a few cases, it is slightly higher under this strategy, though not significantly so. We finally calculated the coefficient of reliability for the single observer between each of capture of the replica assemblages—Supplementary Online Table 3 shows that the $R$ values ranged from 0.994 to 0.999, suggesting that < 1% of the variance in the single observer data is due to an intra-observer error.

**Table 5:** *P*-values from t-tests (difference in mean) and F-tests (difference in variance) comparing the principal component (PC) scores of the repeats of each tool (1–6) captured by multiple observers verses a single observer. Statistical significance (*p* < 0.05) is marked by an asterisk (*). All values have been rounded to 3 decimal places

Tool	PC1		PC2		PC3	
	T	F	T	F	T	F
1	0.282	0.068	0.556	0.141	0.110	0.001*
2	0.091	0.463	0.114	0.162	0.188	0.671
3	0.006*	0.119	0.067	0.873	0.335	0.115
4	0.082	0.029*	0.009*	0.384	0.099	0.006*
5	0.004*	0.663	0.003*	0.257	0.000*	0.411
6	0.954	0.056	0.095	0.157	0.441	0.939

**Table 6:** Summary statistics reporting mean (m) and standard deviation (sd) of principal component (PC) scores of the repeats of each tool (1–6), captured by multiple observers versus a single observer. All values have been rounded to 3 decimal places

Tool	PC1				PC2				PC3			
	Multiple		Single		Multiple		Single		Multiple		Single	
	m	sd	m	sd	m	sd	m	sd	m	sd	m	sd
1	− 0.034	0.006	− 0.031	0.003	0.039	0.007	0.037	0.003	− 0.011	0.016	− 0.023	0.003
2	0.034	0.003	0.031	0.002	0.046	0.002	0.042	0.004	0.042	0.004	0.039	0.004
3	− 0.131	0.001	− 0.135	0.002	− 0.111	0.003	− 0.107	0.004	0.009	0.004	0.007	0.002
4	0.174	0.005	0.169	0.002	− 0.074	0.004	− 0.081	0.003	− 0.004	0.01	− 0.012	0.002
5	− 0.03	0.001	− 0.033	0.002	0.03	0.003	0.025	0.001	− 0.014	0.001	− 0.023	0.002
6	− 0.008	0.007	− 0.008	0.003	0.078	0.002	0.074	0.004	− 0.006	0.006	− 0.004	0.006

## Discussion

Here we present a control study that validates the use of the collaborative data collection protocol presented in Timbrell (2022), which can now be used more extensively by other researchers to reduce travel and carbon emissions, as well as to bring researchers from other geographical areas into the collaborative process more directly. Our results demonstrate that the levels of inter-observer error permeating shape data collated under a collaborative research framework fall within the acceptable threshold, thanks to the establishment of clear research protocols followed by each collaborator. We found that, inevitably, increases in error occur as a consequence of relying on multiple observers, who each have access to different equipment, yet we do not deem this to be significant enough to highly distort the results towards a different conclusion about the data. Therefore, our innovative 3D printing approach and the results reported here have important implications for error assessments of linear metric and GMM data when recording lithic shape as well as the aggregation of data collected by multiple observers.

Outline-based GMM was found to be slightly more sensitive to inter-observer error than metric methods. As Caple et al. (2018) point out, EFA involves global descriptors capturing around 99% of the variance in the outline shape, and therefore, discrepancies between images lead to error in the coefficients dispersed throughout the full outline. Therefore, even if the error is not equally distributed, it is measured as such, and consequently, outline methods are often more sensitive to error than linear methods that capture only certain dimensions of an object. 2D outline-based GMM provides comprehensive morphological information on the gross outline shape of an object, whereas linear metrics are able to capture aspects of the 3D shape but in much less detail; the increase in the morphological information captured, plus the added potential for automated data capture (e.g. Bonhomme et al. 2014; Matzig 2021) and impressive shape visualization (e.g. Figure 5), will be worth the potential increase in error with 2D GMM in many scenarios.

Our use of PCA to highlight axes of variance within lithic shape assemblages also demonstrates that inter-observer error does not affect all PCs equally. As outlined by Page (1976), subtle errors in each variable are combined in multivariate analyses and can be extracted by a single or small set of PCs, although they may also describe real aspects of covariance and so require careful consideration as to their source. When undertaking metric analyses, it is possible to assess error in each individual measurement; if the metrics are combined via dimension reduction methods such as PCA, the contributions of each individual measurement to each PC are readily identifiable through the PCA coefficients. This is less feasible with GMM data, particularly when using outlines and semi-landmarks, and in such cases, it is preferable to assess error on each of the leading PCs, as demonstrated above, rather than on each set of coordinates, which can be very numerous. Overall, the error is impossible to avoid completely, and indeed, the imperfect fidelity of cultural transmission means that copying errors can naturally occur during the knapping process and inflate variance between and within assemblages (Eerkens and Lipo 2005; Schillinger et al. 2014). In this sense, the error is certain to arise within a data set capturing lithic variability; however, steps can be taken to ensure it is minimized, such as standardization of data acquisition, processing, and analytical procedures, calibration, high-quality equipment and assessment of error through repeat measures (Evin et al. 2020; Lyman and VanPool 2009; Robinson and Terhune 2017; Yezerinac et al. 1992). In the case of the current study, we determine that inter-observer error is low enough for accurate analyses under both methods, especially as the high $ICC$ and $R$ values demonstrate acceptable levels of congruence between the six observers.

Through the development of clear research protocols, our results demonstrate that multiple observers can successfully work together to produce sets of comparable data for aggregation. We believe that collaborative research designs, such as the one reported in Timbrell (2022), play an integral role in addressing the vulnerabilities of international research to disruption, revealed most recently in 2020 by the outbreak of coronavirus (COVID-19), which halted both domestic and international travel as well as social interaction. Our results suggest that, as well as single researchers visiting multiple collections to independently access lithic samples, international colleagues are also able to work together in situ to generate data, thereby building resilience in archaeological practice (Douglass et al., 2020; Scerri et al., 2020). We stress though that collaborative research designs should involve an equitable partnership in relation to the data, following the imminent Cape Town statement (see Else, 2022), with all researchers being involved in all stages of the research, from planning and protocol development to publication and dissemination (Chirikure 2015; Douglass et al. 2020). In this way, dual project development can enable local researchers to benefit from international archaeological research, thereby avoiding some (but not all) of the neo-colonial “helicopter” practices that have been hugely criticized in archaeological and anthropological sciences, particularly in Africa (Ackermann 2019; Athreya and Ackermann 2019; Sahle 2021). We have provided here an initial pilot test of collaborative data collection using a 3D printing approach. This approach is unique and, to our knowledge, has not yet been applied in the context of lithic variability nor inter-observer error assessments. We propose that future studies should aim to reproduce our approach with more expanded samples of replica artefacts and discuss three important aspects of potential future study design below.

The first aspect relates to the use of statistics and simple metrics for reporting the inter-observer error. Statistics such as the $ICC$ and %$TEM$ express the error variance relative to the overall variance of the sample; the variance is decomposed into that due to genuine variation among the artefacts and that due to variation among the observers (including that due to different individuals, their different cameras, lenses, etc.). Whilst this approach has many advantages, one immediate drawback is that these statistics are directly affected by the magnitude of genuine variation in both the sample of artefacts and in the dimensions measured. A given constant level of measurement error will appear large when the artefacts measured are highly standardized, but small when the artefacts measured are highly variable. Even if one were to measure the widths and lengths of a set of highly standardized artefacts, a given level of measurement error would appear smaller the further the ratio of width to length is from unity, as this would increase the magnitude of genuine variation in the measurements taken. For this reason, it is always valuable to present simple indices of *absolute* error (such as standard deviation or variance) for *single* measurements alongside the indices of relative error variance across all measurements provided by the $ICC$ and $\%TEM$. Such simple indices are valuable in assessing inter-observer error even when the ultimate study involves more sophisticated morphological analyses, such as those based on GMM. In the current study, Table 2 presents such indices and demonstrates that levels of error are minimal (the largest standard deviation among multiple observers for a single measurement = 0.613 mm).

The second aspect relates to the exploration of the effects of the raw material used for the production of the reference collection on the results of comparative studies. In this study, we used flint because it was available and accessible at the University of Liverpool, where the materials were prepared. This fine-grained raw material tends to produce well-defined features and edges, and so it would be interesting to replicate the approach with a more coarse-grained material, such as quartzite, chert, calcrete or sandstone. This is especially pertinent in our case as the shapes obtained from these materials are likely to be more representative of the actual African stone tools that have been recorded in the main project. However, we note that heat-treated silcrete may achieve a grain as fine as flint (Key et al. 2021), and that obsidian can be even finer-grained than flint; since both silcrete and obsidian are raw materials commonly found in African Middle Stone Age assemblages. We suggest that the flint used here acts as a suitable middle ground in terms of granularity and can therefore be considered as broadly comparable to those raw materials studied in the main project.

Finally, an aspect of variation between individual replicas that we did not explicitly measure is that which can arise through 3D printing. Zeng and Zou (2019) outline some of the factors that can affect the precision of 3D printing, which include slicing and support errors. However, we propose that, even if there are printing errors present in our replicas, these are likely minimal due to the highly comparable data obtained across the project. Additionally, printing errors should not contribute to differences between the two data collection strategies as both the multiple observers and the single observer recorded measurements from the same set of replicas. Depending on the local accessibility of 3D printers, our approach to inter-observer testing could be further streamlined through the direct sharing of the virtual 3D models, with each collaborator printing their own copies to measure. This would alleviate potential logistical problems with global distribution, both via mail or directly, though further research is required to ascertain the variation in objects printed using different models of 3D printers.

## Conclusion

Aggregating lithic shape data requires careful consideration in to order reduce potential sources of inter-observer error that can result in detrimental consequences on the results and their interpretation. Our analysis of metric and outline-based 2D GMM data from multiple observers found that the former performed slightly better than the latter in our tests of inter- and intra-observer error, primarily due to differences in the nature and detail of the morphological information obtained, though both approaches returned levels of error deemed acceptable for accurate analyses. Standardization of the data collection procedure is vital for ensuring that congruence between observers is maintained, though we note that this alone cannot completely eradicate error as we find that variability between observers can still be detected within our data to a (sometimes) significant extent. Nonetheless, we believe that producing replica samples through 3D printing could have many useful applications within archaeological and anthropological sciences beyond the study of error in the analysis of lithic assemblages and should be adopted more widely in assessments of inter-observer error as an integral component of international collaborations between institutions.

## Supplementary Information

Below is the link to the electronic supplementary material.

Supplementary file1 (DOCX 720 KB)

## Data Availability

All data and R code can be found on the project’s repository and was made available for the peer-review of this article: https://github.com/lucytimbrell/error_analysis_lithics/.
