# Supplementary material for: Testing inter-observer error under a collaborative research framework for studying lithic shape variability
Source: Archaeol Anthropol Sci. 2022 Oct 1;14(10):209. doi: 10.1007/s12520-022-01676-2 (PMC9525927; doi:10.1007/s12520-022-01676-2)
Supplement: Supplementary file 1 — Supplementary file1 (DOCX 720 KB) [file 12520_2022_1676_MOESM1_ESM.docx]

**Testing inter-observer error under a collaborative research framework for studying lithic shape variability –** Archaeological and Anthropological Sciences

Lucy Timbrell^1*^, Christopher Scott^1^, Behailu Habte^2^, Yosef Tefera^2^, Hélène Monod^3^, Mouna Qazzih^4^, Benjamin Marais^5^, Wendy Black^5^, Christine Maroma^6^, Emmanuel Ndiema^6,7^, Struan Henderson^8^, Katherine Elmes^8^, Kimberly Plomp^9,10^ and Matt Grove^1^

^1^ Department of Archaeology, Classics and Egyptology, University of Liverpool, Liverpool, United Kingdom

^2^ Authority for Research and Conservation of Cultural Heritage, National Museum of Ethiopia, Addis Ababa, Ethiopia

^3^ Département Homme et Environnement, Musée de l’Homme, Paris, France

^4^ Institut National des Sciences de l’Archéologie et du Patrimoine, Rabat, Morocco

^5^ Archaeology Unit, Iziko Museums of South Africa, Cape Town, South Africa

^6^ Department of Archaeology, National Museums of Kenya, Nairobi, Kenya

^7^ Max Planck Institute for the Science of Human History, Jena, Germany

^8^ Mossel Bay Archaeological Project, Western Cape Province, South Africa

^9^ Archaeological Studies Program, University of the Philippines, Quezon City, Philippines

^10^ Department of Archaeology, Simon Fraser University, British Colombia, Canada

* Corresponding Author: Lucy Timbrell ([lucy.timbrell@liverpool.ac.uk](mailto:lucy.timbrell@liverpool.ac.uk)), Department of Archaeology, Classics and Egyptology, University of Liverpool, Liverpool, United Kingdom

**Supplementary Online Resource S1: Description of photography protocols**

This section summarises the standardised photography procedure outlined in Timbrell (preprint). These protocols were followed by all observers to regulate the photography set-up and data capture.

*Artefact position:* All tools were positioned so that the equivalent surface was facing the camera. The artefacts were secured to a contrasting background (often white coloured) and levelled so that the outlines were homologous. A scale was positioned in the same plane as the artefact and far enough away from the tool so that the outline could be easily generated.

*Camera position and settings:* The back of the camera was positioned at a set distance away from the tool using a copy stand or tripod. The camera was levelled and recalibrated to ensure it remained flat for the duration of the research. Distortion settings on the camera were turned off and aperture priority mode turned on. Remote capture systems, such as computer software, digital remotes, and built-in timers, were employed to minimise camera movement.

*Lighting:* Lights were positioned to minimise shadows around the edge of the tool. When available, a light box was employed.


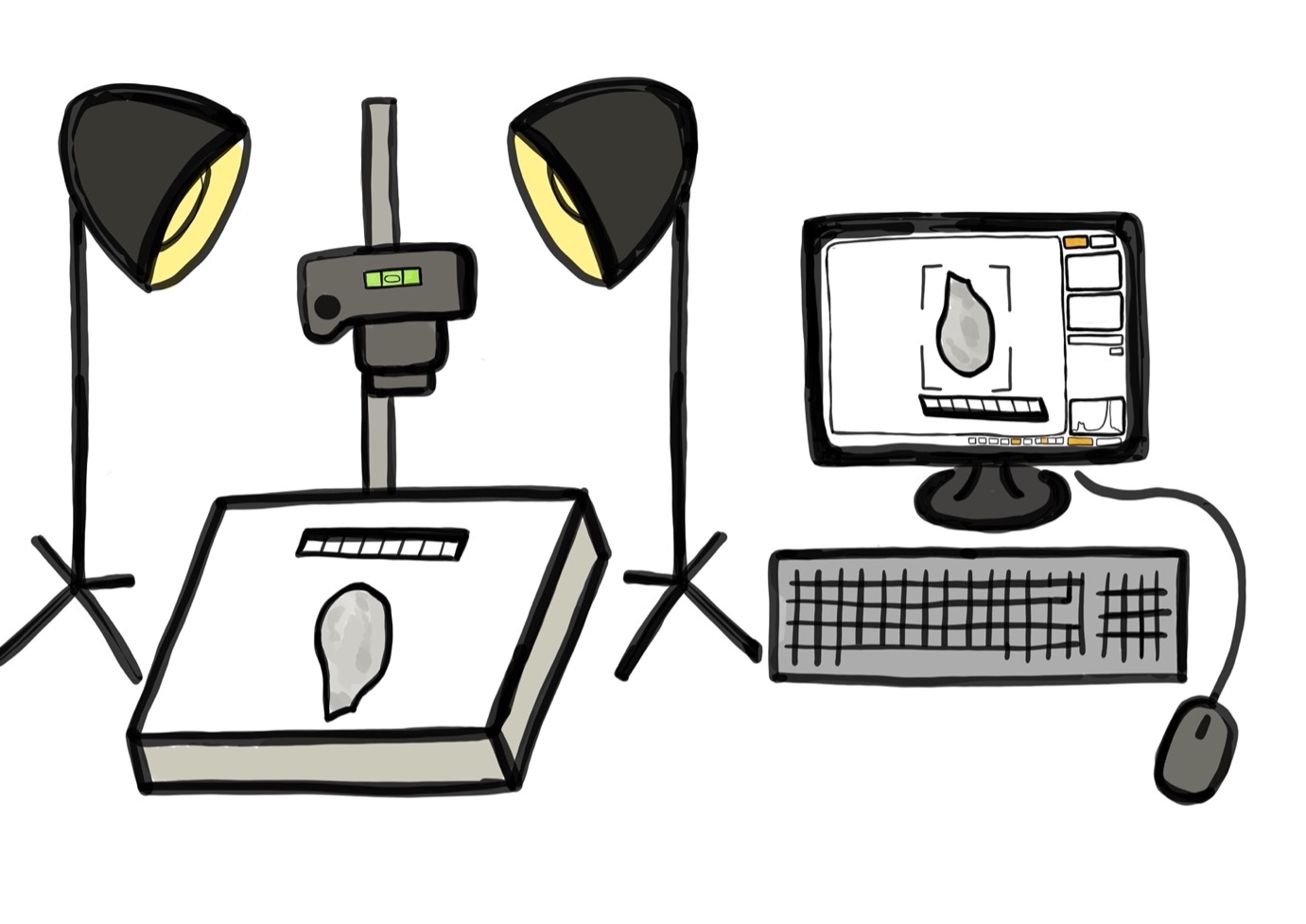
*Data recording:* Images were saved as .jpeg files. A spreadsheet was provided to record the file names of each photo with the metric measurements. Data was uploaded onto a communal data storage platform in preparation for processing and analysis.

**Supplementary Online Resource S1 Fig. 1** A schematic of the photography set-up, reprinted from Timbrell (under review).

**Supplementary Online Table 1** Triangle and vertices data for the obj. models that were created fro 3D printing.

| Model | 1 | 2 | 3 | 4 | 5 | 6 |
| --- | --- | --- | --- | --- | --- | --- |
| Triangles | 4739974 | 6511000 | 3979922 | 9021498 | 3658156 | 8977254 |
| Vertices | 14219922 | 19533000 | 11939766 | 27064494 | 10974468 | 26931762 |

**Supplementary Online Table 2** Tukey HSD results comparing artefact group means of principal components (PC) 1-3. Statistical significance is marked as p < 0.05 (*) and < 0.001 (**).

| **Artefact number** | **PC1** | **PC2** | **PC3** |
| --- | --- | --- | --- |
| 2-1 | 0.000** | 0.292 | 0.000** |
| 3-1 | 0.000** | 0.000** | 0.017* |
| 4-1 | 0.000** | 0.000** | 0.968 |
| 5-1 | 0.676 | 0.008* | 0.999 |
| 6-1 | 0.000** | 0.000** | 0.980 |
| 3-2 | 0.000** | 0.000** | 0.000** |
| 4-2 | 0.000** | 0.000** | 0.000** |
| 5-2 | 0.000** | 0.000** | 0.000** |
| 6-2 | 0.000** | 0.000** | 0.000** |
| 4-3 | 0.000** | 0.000** | 0.000** |
| 5-3 | 0.000** | 0.000** | 0.101 |
| 6-3 | 0.000** | 0.000** | 0.007* |
| 5-4 | 0.000** | 0.000** | 0.088 |
| 6-4 | 0.000** | 0.000** | 1 |
| 6-5 | 0.000** | 0.000** | 0.886 |

**Supplementary Online Table 3** Coefficient of reliability (R) values for pair-wise combinations of assemblages using the first 3 PC scores, recorded by the single observer. For assemblage numbers and associated observer codes, see Table 1. All values have been rounded to 3 decimal places.

|  | **Assemblage 1** | **Assemblage 2** | **Assemblage 3** | **Assemblage 4** | **Assemblage 5** |
| --- | --- | --- | --- | --- | --- |
| **Assemblage 2** | 0.998 |  |  |  |  |
| **Assemblage 3** | 0.998 | 0.998 |  |  |  |
| **Assemblage 4** | 0.997 | 0.998 | 0.995 |  |  |
| **Assemblage 5** | 0.996 | 0.995 | 0.994 | 0.996 |  |
| **Assemblage 6** | 0.998 | 0.997 | 0.999 | 0.995 | 0.995 |


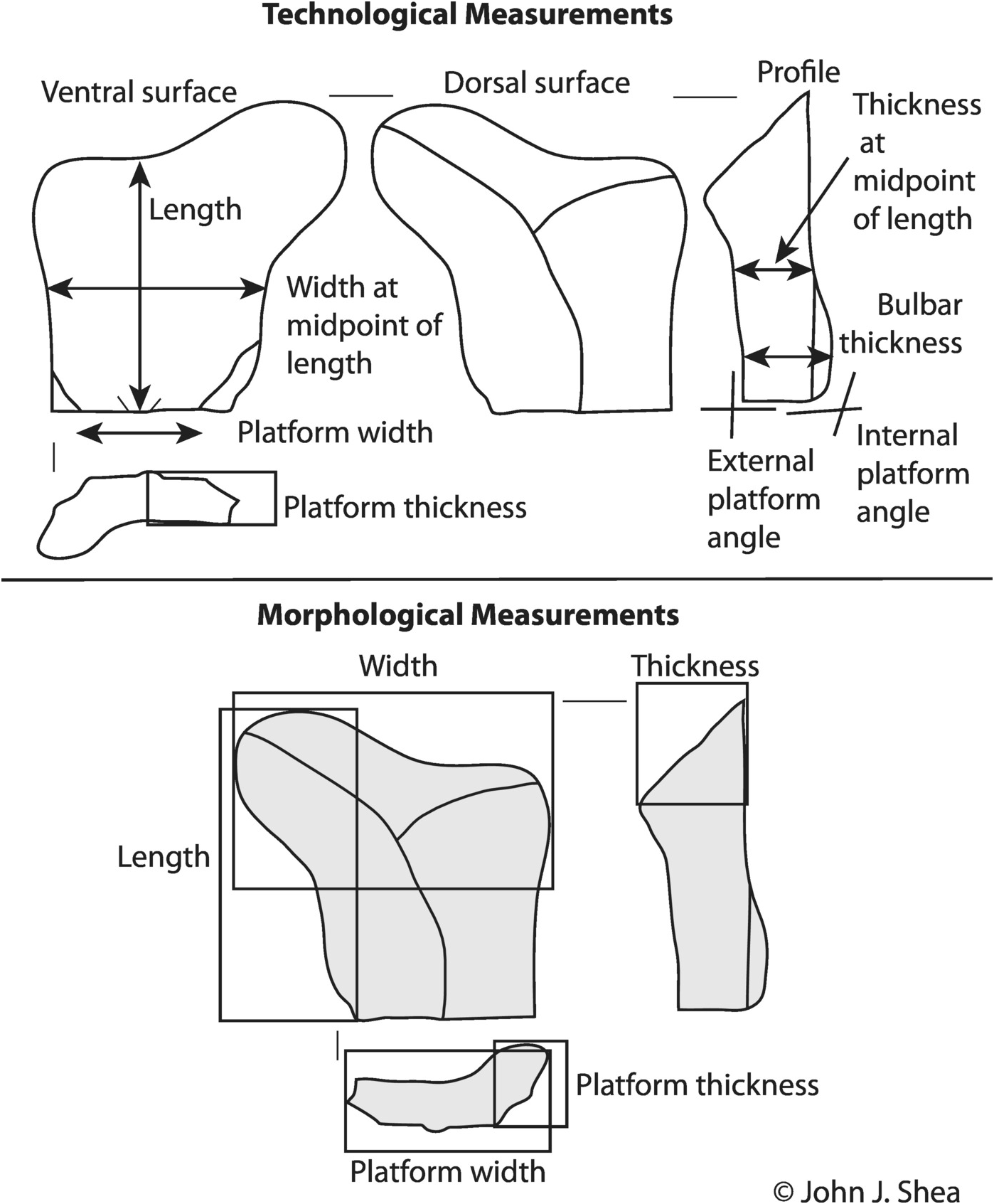


**Supplementary Online Fig. 1** A schematic reprinted from Shea (2020) demonstrating the morphological measurements recorded. Length is defined as the maximum dimension of the lithic, width as the maximum measurement in the perpendicular dimension to length, and thickness as the maximum measurement in the third dimension


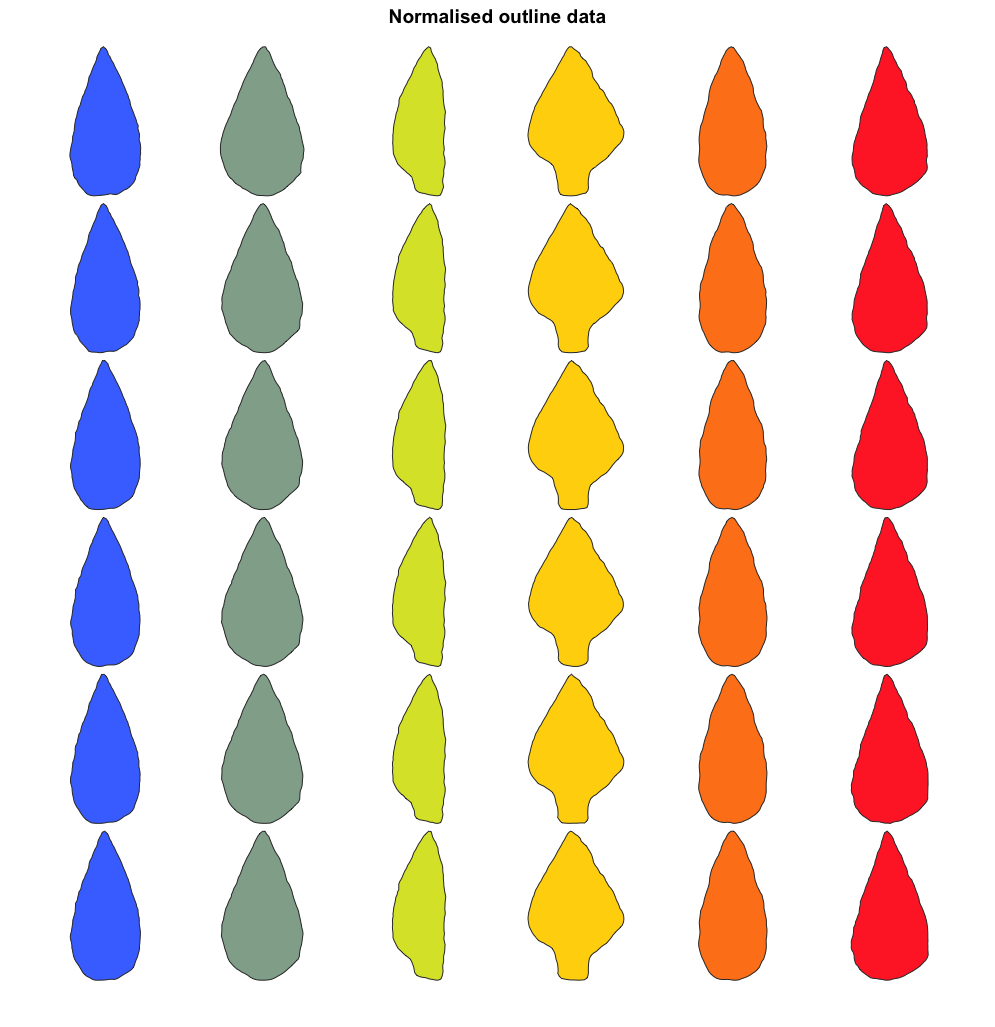
**Supplementary Online Fig. 2** Outline data of the tools captured by six observers. Replicas are coloured by tool type.


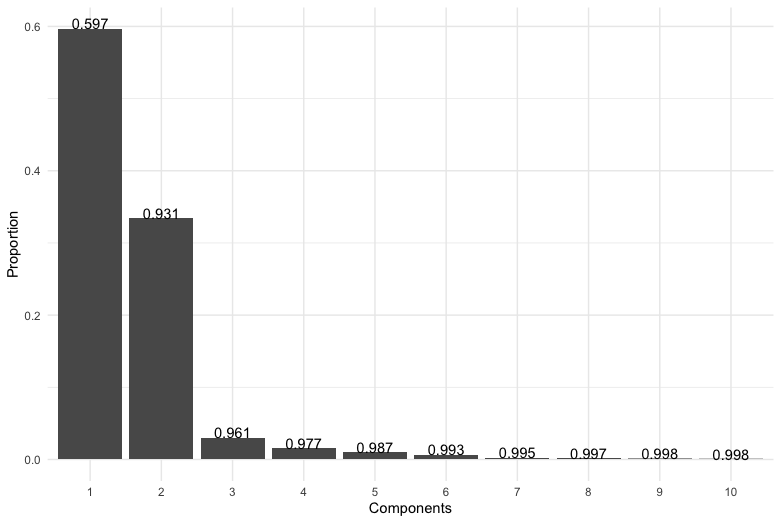


**Supplementary Online Fig. 3** Scree plot of the principal component (PC) loadings of the multiple observer data, with the cumulative variance reported above the bars.


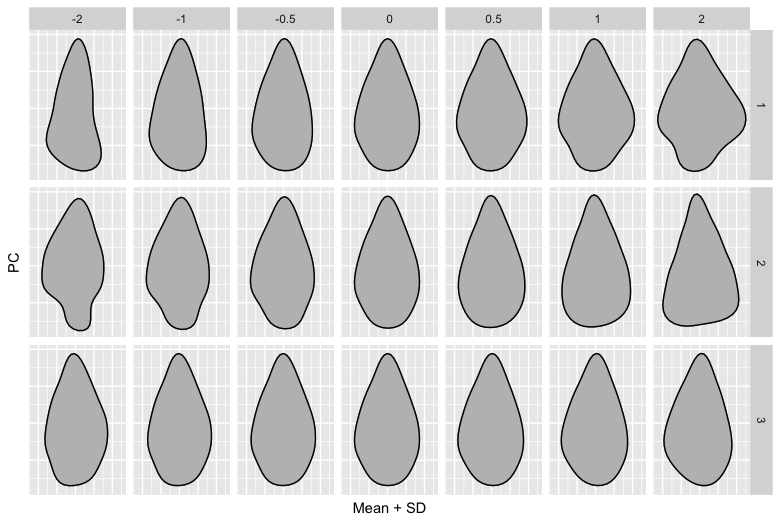


**Supplementary Online Fig. 4** Principal component (PC) contributions along the first 3 axes for both the single and multiple observer data. Note this is very similar to Figure 5, as is to be expected.


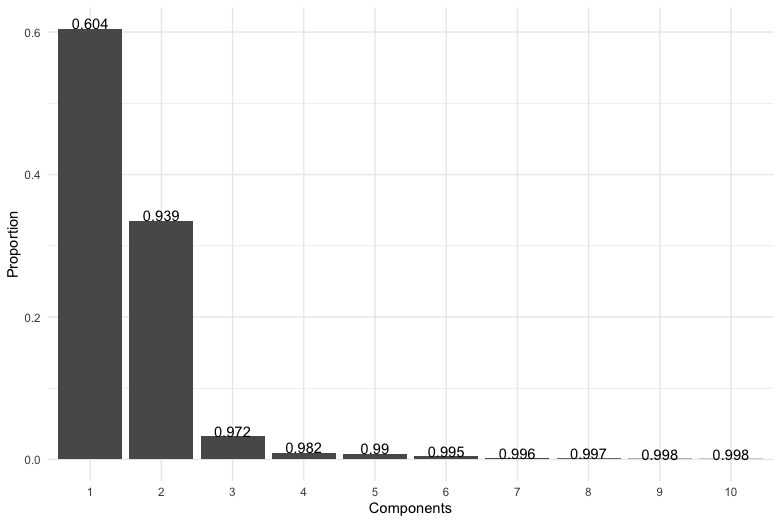


**Supplementary Online Fig. 5** Scree plot of the principal component (PC) loadings of the single and multiple observer data together, with the cumulative variance reported above the bars.
